# Pharmacokinetics and relative bioavailability study of two cefquinome sulfate intramammary infusions in cow milk

**DOI:** 10.3389/fvets.2024.1384076

**Published:** 2024-03-11

**Authors:** Shuang Li, Na Yu, Yaoxin Tang, Chunshuang Liu, Ying Zhang, Xiaojie Chen, Hao Wu, Xiubo Li, Yiming Liu

**Affiliations:** ^1^National Feed Drug Reference Laboratories, Feed Research Institute, Chinese Academy of Agricultural Sciences, Beijing, China; ^2^Tianjin Key Laboratory of Agricultural Animal Breeding and Healthy Husbandry, College of Animal Science and Veterinary Medicine, Tianjin Agricultural University, Tianjin, China; ^3^Key Laboratory of Animal Antimicrobial Resistance Surveillance, Ministry of Agriculture and Rural Affairs, Feed Research Institute, Chinese Academy of Agricultural Sciences, Beijing, China; ^4^Laboratory of Quality and Safety Risk Assessment for Products on Feed-origin Risk Factor, Ministry of Agriculture and Rural Affairs, Feed Research Institute, Chinese Academy of Agricultural Sciences, Beijing, China; ^5^College of Veterinary Medicine, China Agricultural University, Beijing, China

**Keywords:** pharmacokinetics, relative bioavailability, UPLC-MS/MS, cefquinome sulfate intramammary infusion, cow milk

## Abstract

In this study, two intramammary infusions of cefquinome sulfate were investigated for pharmacokinetics and bioavailability. Twelve lactating cows for each group were administered an effective dose of 75 mg/gland for cefquinome, with milk samples collected at various time intervals. The concentrations of cefquinome in milk at different times were determined by the UPLC-MS/MS method. Analyses of noncompartmental pharmacokinetics were conducted on the concentration of cefquinome in milk. Mean pharmacokinetic parameters of group A and group B following intramammary administration were as follows: AUC_last_ 300558.57 ± 25052.78 ng/mL and 266551.3 ± 50654.85 ng/mL, C_max_ 51786.35 ± 11948.4 ng/mL and 59763.7 ± 8403.2 ng/mL, T_1/2_ 5.69 ± 0.62 h and 5.25 ± 1.62 h, MRT 7.43 ± 0.79 h and 4.8 ± 0.78 h, respectively. Pharmacokinetic experiments showed that the relative bioavailability of group B was 88.69% that of group A. From our findings, group B (3 g: 75 mg) shows a quicker drug elimination process than group A (8 g: 75 mg), which suggests that the withdrawal period for the new formulation may be shorter.

## 1 Introduction

Mastitis, a prevalent affliction affecting cows, exerts an adverse influence on both the well-being of animals and the financial viability of farms (1). Previous research has shown that the economic losses suffered by farms vary from $1.20 per cow per day in the first month to $2.06 per cow per day in the 10th month (2). *Staphylococcus aureus* and *Escherichia coli* account for 40 ~ 50% of dairy foods, which poses a risk to human health (3–5). Antibiotic therapy accounts for a large proportion of mastitis treatment, including marbofloxacin, penicillin, rifaximin, cefquinome, etc., which can be administered through intramuscular/intravenous or intramammary injection (6–8). In order to attain high bioavailability, which is difficult to achieve through other administration routes, antibiotics are typically administered by intravenous injection (9). Moreover, the extensive systemic treatment and control of cow mastitis does not consistently yield desirable efficacy (10). The local antibiotics administrated through intramammary infusion effectively treat pathogens infections by enhancing the drug concentration at the site of administration (11, 12). Recently, there has been a noticeable trend towards the development of intramammary infusion in the field of mastitis treatment. Previous research indicates that intramammary infusions accounted for 81% of the total products used on farms in Ireland (13). Also, approximately 85.4% of intramammary infusions were employed in Argentina aimed at treatment of disease (14).

**Figure 1:**
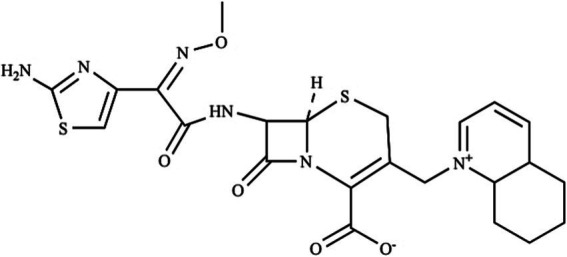
The chemical structural formula of cefquinome.

Cefquinome, the cephalosporin of the fourth generation, which has a broad spectrum of antimicrobial activity (15). The chemical structural formula of cefquinome is shown in Figure 1. In contrast to second and third generation cephalosporins, chemical modification of the basic structure of cephalosporins provides the amphoteric nature of cefquinome, which can promote rapid penetration of biological membranes and improve bioavailability (16, 17). Cefquinome is a veterinary specific drug that is used in veterinary clinics for the treatment of respiratory diseases in horses (18) and pneumonia in dogs (19). In dairy farming, it is widely used for the treatment of cow’s under and respiratory infections, as well as the treatment of cow’s endometritis (20, 21). A European multicentre study showed high *in vitro* activity of cefquinome against a broad spectrum of cow’s pathogens (22).

Previous studies showed that the commercial cefquinome sulfate intramammary infusion had good pharmacokinetic characteristics in dairy cows. Xiao et al. (23) also studied the pharmacokinetic characteristics of 75 mg cefquinome intramammary infusion in the milk of healthy dairy cows. The results showed that cefquinome eliminated slowly and the effective concentration in milk maintained for a long time. Li et al. (24) investigated the pharmacokinetics of cefquinome in plasma and milk samples of lactating cows following a dose of 75 mg, and the results showed that cefquinome did not penetrate blood and was eliminated rapidly from milk after intramuscular administration in lactating cows.

The irrational use of antibiotics in veterinary clinics has led to adverse effects such as bacterial resistance and allergic reactions (25). Previous research has indicated that bacterial strains isolated from milk samples collected across eleven provinces in China exhibit multifaceted antibiotic resistance (26). It is assumed that antimicrobial resistance poses a health risk to humans through the introduction of resistant pathogens into the food chain and the horizontal spread of resistance determinants to other bacteria (27). The presented cases illustrate that irrational dosing leads to drug failure to achieve therapeutic effects. In response to this issue, an improved antibiotic dosing regimen could ensure the effectiveness of the entire treatment process (28). The fact that the utilization of pharmacokinetics for the purpose of designing dosing regimens is not a new phenomenon, it is crucial for pharmaceutical developers to identify the most effective dosage regimen through pharmacokinetics (29, 30).

The effective, stable, and safe treatment of diseases heavily relies on the development of pharmaceutical formulations, which primarily necessitates costly and time-consuming clinical trials (18, 19). In an environment with limited resources, specific development trajectories and controllable composition can be utilized to optimize each new formula (31). The main challenge with intramammary delivery is delivering relatively high doses within a limited volume to minimize discomfort or adverse side effects (32). Therefore, a new intramammary infusion of cefquinome sulfate (3 g: 75 mg) was developed, which has a higher effective concentration of the drug per unit volume of intramammary infusion as compared to the previous size (8 g: 75 mg). The new cefquinome sulfate intramammary infusion contains a 62.5% decrease in the weight over the previous one.

This study investigated the pharmacokinetics of two cefquinome intramammary infusions and obtained the pharmacokinetic parameters. As well as relative bioavailability was obtained by the area under curve (AUC) method. It will provide a scientific basis for dairy farmers to use the infusions in clinical practice, thus reducing the adverse outcomes resulting from irrational use.

## 2 Materials and methods

### 2.1 Reagents

The referential substance of cefquinome sulfate (with an assay purity of 82.6%, batch number: K0321406) was procured from the China Institute of Veterinary Drug Control situated in Beijing, China. The group A: Cefquinome Sulfate Intramammary Infusion (Lactating Cow) (8 g: 75 mg, batch number: 120215), provided by Huaqinyuan Animal Pharmaceutical Co., Ltd. The group B: Cefquinome Sulfate Intramammary Infusion (Lactating Cow) (3 g: 75 mg, batch number: 210910001), provided by Zhongmu Nanjing Animal Pharmaceutical Co., Ltd., (not commercially available). Ultrapure water was prepared by using the Cascada I PALL equipment from New York, United States. Methanol and Acetonitrile of liquid chromatography/mass spectrometer grade used in this study were obtained from Fisher Scientific in Fair Lawn, NJ, United States. Other reagents used in this study were analytical grade and obtained from suppliers in Beijing, China.

### 2.2 Instruments

Waters ACQUITY UPLC ultra-performance liquid chromatograph, by Waters Corporation, United States. Triple quadrupole mass spectrometer, Xevo TQ-S, by Waters Corporation, United States. Analytical balance, CPA225D, by Sartorius, Germany. Solid phase extraction device by Waters Corporation.

### 2.3 Animals

Twelve lactating cows of each group, with an approximate age of 36 months and a weight range of 600 ± 50 kg, were from a commercial dairy farm in Changping District, Beijing. Throughout the research period, cows were healthy and had unrestricted access to water and a standard antibiotic-free diet. Procedures involving animals in the experiment were conducted accordance with the guidelines, which was approved by the Animal Use and Care Committee of the Feed Research Institute, Chinese Academy of Agricultural Sciences (IFR-CAAS20230309). Cows were healthy after the entire experiment and no anesthesia and euthanasia were involved.

### 2.4 Sample collection

For group A (8 g: 75 mg) and group B (3 g: 75 mg), one tube of injectant with 75 mg of cefquinome was administered intramammary to the right front gland of cow. Massage about 30 s to spread the infusion homogeneously. Blank samples were collected as controls from each gland before administration. After single dose administration, milk samples of 20 mL were then collected at 0.25 h, 0.75 h, 1 h, 2 h, 4 h, 6 h, 8 h, 12 h, 24 h, 30 h, 36 h, 48 h, 54 h, 60 h, and 72 h. For the untreated gland (left rear), milk samples of 20 mL were collected at 2 h, 6 h, 12 h, and 24 h after administration. The specimens of milk were preserved in a state of deep freeze at negative 40°C pending further analysis (24).

### 2.5 Sample preparation

One gram of milk sample was delicately transferred into centrifuge tube (10 mL), followed by the addition of 4 mL of acetonitrile. The mixture was vortexed for 3 min and shaken for an additional 10 min, then centrifuged at approximately 7,656 ×*g* for 10 min at 4°C. Next, the sample solution was dried with nitrogen gas at 40°C. The resultant precipitate was reformed into 3 mL solution comprising aqueous formic acid and acetonitrile in a volumetric ratio of 95: 5. The sample solution was passed through a solid-phase extraction (SPE) cartridge (HLB, 60 mg/3 mL) pre-activated with 3 mL methanol and water, respectively. The sample solution flows through the SPE extraction cartridge under the action of gravity, followed by eluting the cartridge with 3 mL methanol of milliliters. Next, the nitrogen drying process was repeated. Dissolve the precipitate with 1 mL aqueous formic acid and acetonitrile. Finally, the sample solution was collected into small-volume sample vial after filtration through 0.22 μm syringe filter, and the sample was subsequently analyzed using UPLC-MS/MS.

### 2.6 UPLC-MS/MS conditions

The parameters for the UPLC analysis were configured in the following manner. In the UPLC system, the separation process was carried out using a Waters BEH C_18_ column (50 mm by 2.1 mm by 1.7 μm). Chromatographic separations were performed with an elution system comprising methanol as the organic solvent (phase A) and 0.1% v/v formic acid in water as the aqueous solvent (phase B). The elution process was conducted with unvarying fluid velocity, set precisely at 0.35 milliliters per minute, employing a protocol of linear gradation: from an initial to 0.9 min duration 95% of phase B was used, which was altered to 50% in the next 0.9 to 3 min slice; subsequently for the 3 to 5 min span, it was changed to 10% phase B; and finally, in the last interval from 5 to 6 min 95% of phase B was restored. The entire procedure was conducted sustaining a column temperature of 40 ± 0.5°C. For a more precise description of operating conditions under the positive ionization mode (ESI^+^) (Table 1). The parameters for multiple reaction monitoring (MRM) of cefquinome (Table 2).

**Table 1:** MS parameters.

Parameter	Settings
Ionization mode	Electrospray ionization (positive mode)
Capillary voltage	2.0 kV
Desolvation temperature	350°C
Cone gas flow	350 L/h
Desolvation gas flow	700 L/h
Secondary collision gas	Ar_2_

**Table 2:** MRM parameters.

Precursorion (m/z)	Production (m/z)	Cone Voltage (V)	Collision Energy (eV)
529.2	134.1	34	14
529.2	396.0	34	14

### 2.7 Method validation

The validation of methods holds significant importance in the development of methods, as it entails the establishment of analytical prerequisites and the verification of the method’s performance capabilities to ensure alignment with said prerequisites (33). The method validation process refers to the requirements of Commission Implementing Regulation (EU) 2021/808. The criteria assessed included selectivity, matrix effect (ME), the limit of detection (LOD), the limit of quantifications (LOQ), linearity, accuracy, precision, and stability.

#### 2.7.1 Selectivity and matrix effect

The juxtaposition of chromatograms derived from blank milk and milk samples infused with cefquinome serves as method for appraising the selectivity (34). The evaluation of the matrix effect was ascertained by contrasting the peak area of the blank matrix standard solution (X) against that of the solvent standard solution (Y) (35). A positive numeric represents signal augmentation, whereas a negative attribute denotes signal decrement. When the matrix effect stands at 0%, this implies minimal matrix interference. When the matrix effect’s absolute magnitude is <20%, it’s reasonable to consider matrix impact negligible. Matrix effect becomes moderate when its absolute magnitude resides between 20 and 50%. However, when the absolute magnitude of the matrix effect surpasses 50%, it equates to intense matrix interference (36).

#### 2.7.2 LOD and LOQ

LOD and LOQ were verified by injecting a standard working solution of suitable concentration into the blank milk matrix to prepare samples with various added concentrations. LOD is characterized as the smallest measurable concentration of an analyte in a sample, eliciting a signal-to-noise (S/N) ratio exceeding three. LOQ is denoted as the minimal concentration of the analyte that can result in a signal-to-noise (S/N) ratio surpassing 10.

#### 2.7.3 Linearity

The linear regression analysis was determined by preparing standard solutions of cefquinome at concentrations of 0.2, 1, 5, 10, 25 and 50 ng/g to construct matrix calibration curves. Upon graphing the peak area corresponding to the quantifying ion of the analyte on the y-axis against the concentration of the standard solution on the x-axis, the regression equation and correlation coefficient were calculated. The standard curve method was used to determine the unknown samples by diluting the milk samples with blank milk matrix to bring the concentration within the range of the standard curve.

#### 2.7.4 Accuracy and precision

Drawing upon the recovery rate and variability coefficient garnered from milk spiking, the accuracy and precision were evaluated. Four concentrations (0.2, 10, 20 and 40 ng/g) of blank milk were spiked to determine recovery rates. Analyzing six replicates of QC concentrations within the same day and evaluating five measurements of QC concentrations over five consecutive days assessed the accuracy and precision of the UPLC-MS/MS method.

#### 2.7.5 Stability

The stability of cefquinome was evaluated under varied storage conditions. Cefquinome stock standard solution was first stored at −40°C to assess its stability over a 30 day period. Exposure to three freeze–thaw cycles, transitioning from room temperature to −40°C, was utilized to determine freeze–thaw stability. Long-term stability testing involved storage of these samples at −40°C for an extended duration of 90 days. Lastly, the samples’ stability during residence in the autosampler tray was analyzed at 4°C for 8 h.

### 2.8 Pharmacokinetic parameter analysis

Using Phoenix software (version 8.1, Pharsight, United States), analyze the relationship between milk concentration and time in each cow. Parameter analysis was performed using non-compartmental models, and the major pharmacokinetic parameters of two groups were compared. The pharmacokinetic parameters were presented as the mean plus or minus the standard deviation (SD). Based on previous research, the calculation formula for relative bioavailability (RBA) is as follows:


$$RBA=\frac{{AUC}_{\mathrm{last}}\left(\mathrm{group}\;B\right)}{{AUC}_{\mathrm{last}}\left(\mathrm{group}\;A\right)}\times 100\%$$

## 3 Results

### 3.1 Selectivity and matrix effect

**Figure 2:**
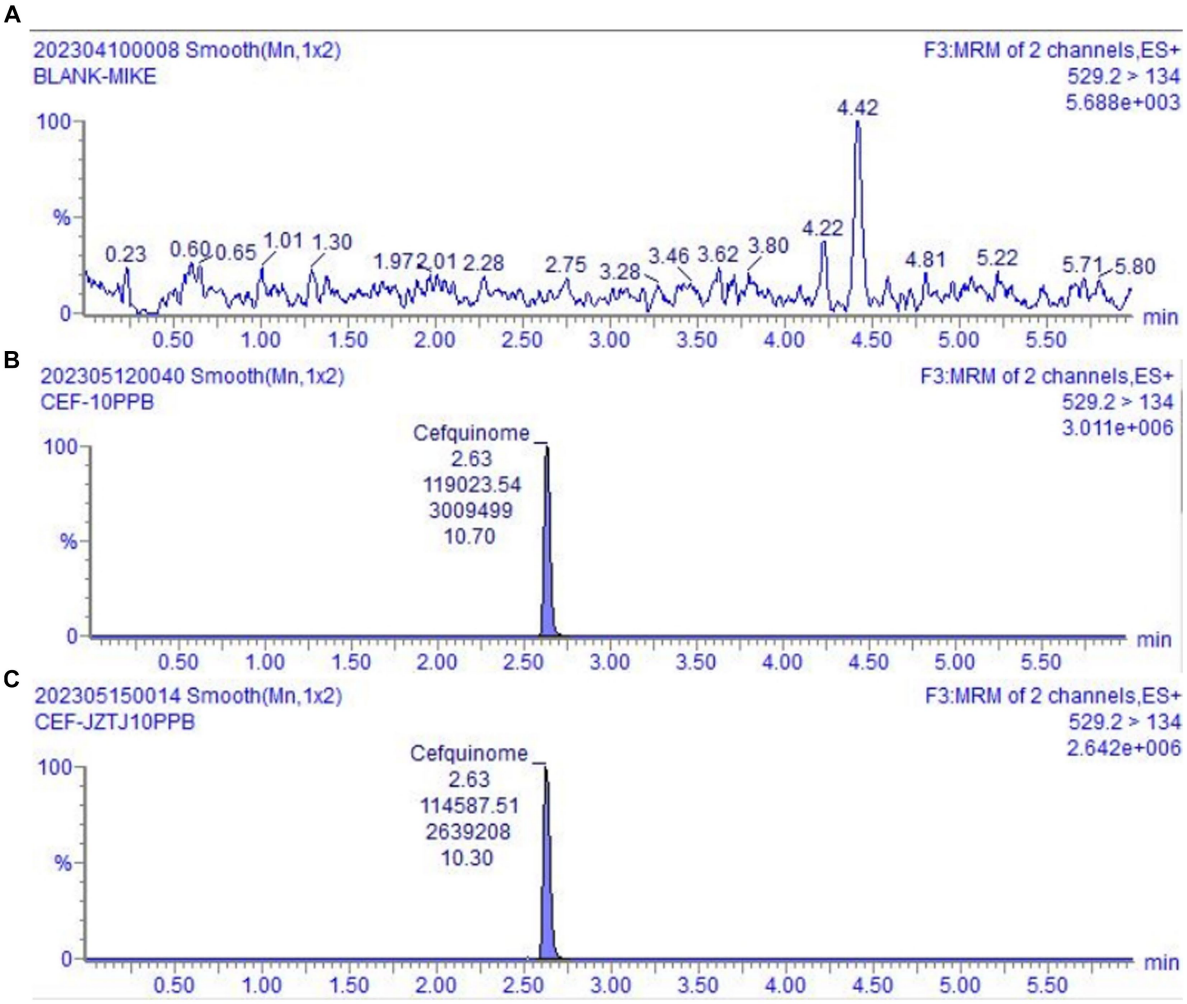
UPLC-MS/MS chromatograms. **(A)** The chromatogram of blank milk sample. **(B)** The chromatogram of blank milk sample added cefquinome (10 ng/g). **(C)** The chromatogram of blank mobile phase added cefquinome (10 ng/mL).

Endogenous substances in blank cow milk do not interfere with the measurement of the target compound being tested, which validated the specificity of the testing approach. The chromatogram depicting blank milk, cefquinome spiked blank milk, and cefquinome spiked blank mobile phase are shown in Figure 2. Matrix effect results showed that there was signal augmentation at milk matrix addition concentrations of 0.2 ng/g and 40 ng/g, and signal decrement at 10 ng/g and 20 ng/g (Table 3). The matrix effect at the milk matrix spiked concentration of 0.2 ng/g was 37.88%, which is considered a medium matrix effect, while the absolute values of the other spiked concentrations are all <20%, categorized as weak matrix effects. The quantification of cefquinome was performed using matrix calibration curve to compensate for matrix effects.

**Table 3:** The matrix effect of cefquinome in milk.

Spiked concentration (ng/g)	0.2			10			20			40		
Batch	1	2	3	1	2	3	1	2	3	1	2	3
ME (%)	20.35	43.67	49.62	−5.73	−11.88	2.15	−15.56	−17.05	−15.64	0.12	4.37	−3.62
Average ME(%)	37.88			−5.15			−16.08			0.29		

### 3.2 LOD and LOQ

**Figure 3:**
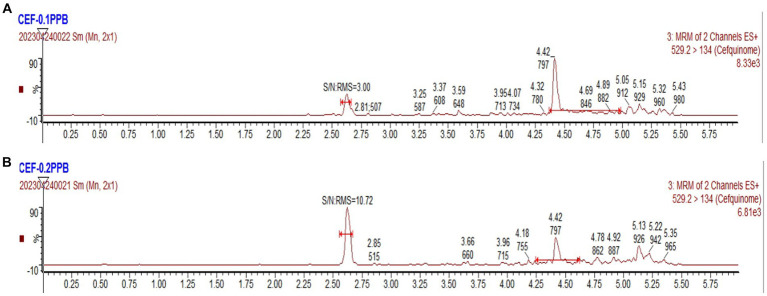
UPLC-MS/MS chromatograms. **(A)** The chromatogram of LOD for cefquinome. **(B)** The chromatogram of LOQ for cefquinome.

With a signal-to-noise ratio (S/N) of ≥3, the detection limit (LOD) value was established at 0.1 ng/g. With an S/N of ≥10, the limit of quantification (LOQ) value was established at 0.2 ng/g. The corresponding chromatograms can be seen in Figure 3.

### 3.3 Linearity

**Figure 4:**
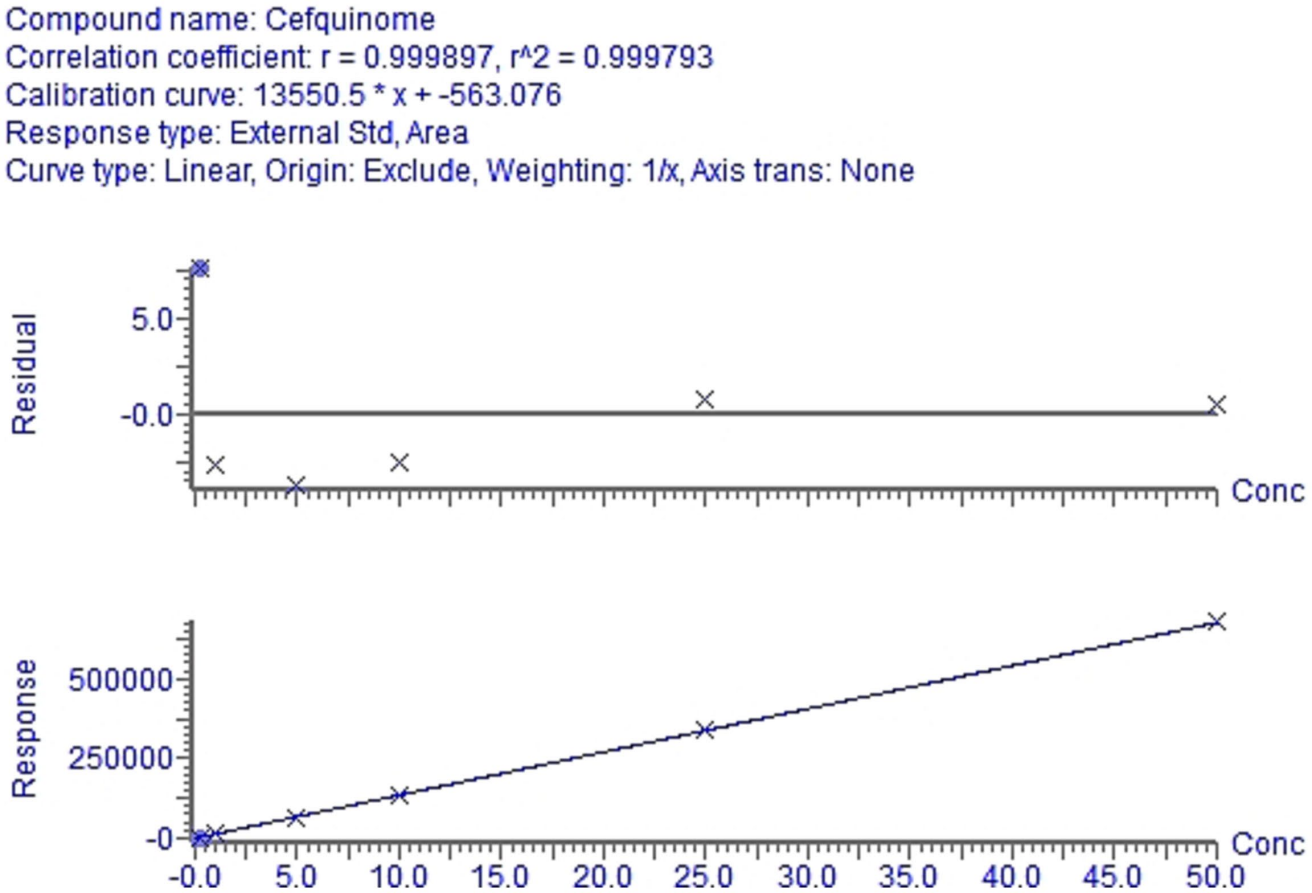
Calibration curve for cefquinome in cow milk.

Based on the weighted (1/x^2^) least-square linear regression analysis results, the matrix calibration curve for cefquinome highlighted the proportional correlation between the cefquinome concentration and the analytical response. The linear relationship was confirmed across the concentration spectrum of 0.2 ~ 50 ng/g and the coefficient of correlation (*r*), surpassing 0.999 (Table 4), revealed a robust correlation between the cefquinome concentration and its corresponding peak intensity. The equations describing this calibration, with X denoting concentration and Y denoting peak area, are provided in Figure 4.

**Table 4:** The calibration equation and correlation coefficients for cefquinome.

Regression equation	Linearity (ng/g)	Correlation coefficient
Y = 13550.5x-563.076	0.2 ~ 50	*r* = 0.999897

### 3.4 Accuracy and precision

Relative standard deviation and recovery were calculated for both intra-day and inter-day variations by performing six parallel tests on each concentration, repeated 5 days. The results are shown in Table 5. The recovery rates of cefquinome in milk have been measured to be between 97.42–99.94%, the intra-batch variation coefficients have been found to be <9.97% and the inter-batch variation coefficient has been determined to be <5.95%. Based on the results of the study, the use of the analytical method employed resulted in accurate and precise quantification of cefquinome in milk samples, satisfying the requirements for a reliable method.

**Table 5:** The results of accuracy and precision for cefquinome in milk at four distinct spiked concentrations. (day = 5, *n* = 30).

Spiked concentration (ng/g)	Average recovery (%)					SD (%)	Intra-day RSD (%)					Inter-day RSD (%)
	1	2	3	4	5		1	2	3	4	5	
0.2	97.90	95.08	98.75	99.21	98.62	5.83	3.61	9.97	4.40	6.57	4.55	5.95
10	94.97	100.00	104.93	99.32	100.48	5.16	4.98	2.89	6.65	2.87	2.59	5.17
20	94.08	97.08	97.98	98.96	99.10	3.73	2.48	4.26	3.37	4.99	2.93	3.95
40	96.99	97.07	95.92	98.18	99.38	3.27	4.25	4.37	2.03	2.87	2.66	3.35

### 3.5 Stability

The stability assessment of cefquinome incorporated a variety of tests: stock standard solution, three freeze–thaw, sample tray, and long-term stability assays (Table 6). After being subjected to three freeze–thaw cycles ranging from −40°C to room temperature, cefquinome maintained its stability in milk. It also exhibited steady characteristics after 8 h storage in a sample tray at 4°C, and a prolonged 90 day storage at −40°C. Moreover, cefquinome stock solutions remained stable over a span of 30 days at −40°C. The results indicate that the above conditions did not seem to affect the quantification of cefquinome.

**Table 6:** Stability of cefquinome under various storage conditions.

Spiked concentration (ng/g)	reserve solution (30 days at −40°C)	Freeze–thaw	Sample tray (8 h at 4°C)	Long-term stability (90 days at −40°C)
	Mean ± SD	Mean ± SD	Mean ± SD	Mean ± SD
0.2		0.21 ± 0.02	0.21 ± 0.01	0.20 ± 0.02
10	10.32 ± 6.67	8.35 ± 0.25	10.32 ± 0.52	8.5 ± 0.16
20		19.71 ± 1.04	19.85 ± 1.06	20.56 ± 0.88
40		38.82 ± 1.94	40.19 ± 1.64	39.92 ± 2.18

### 3.6 Pharmacokinetic parameter analysis

**Figure 5:**
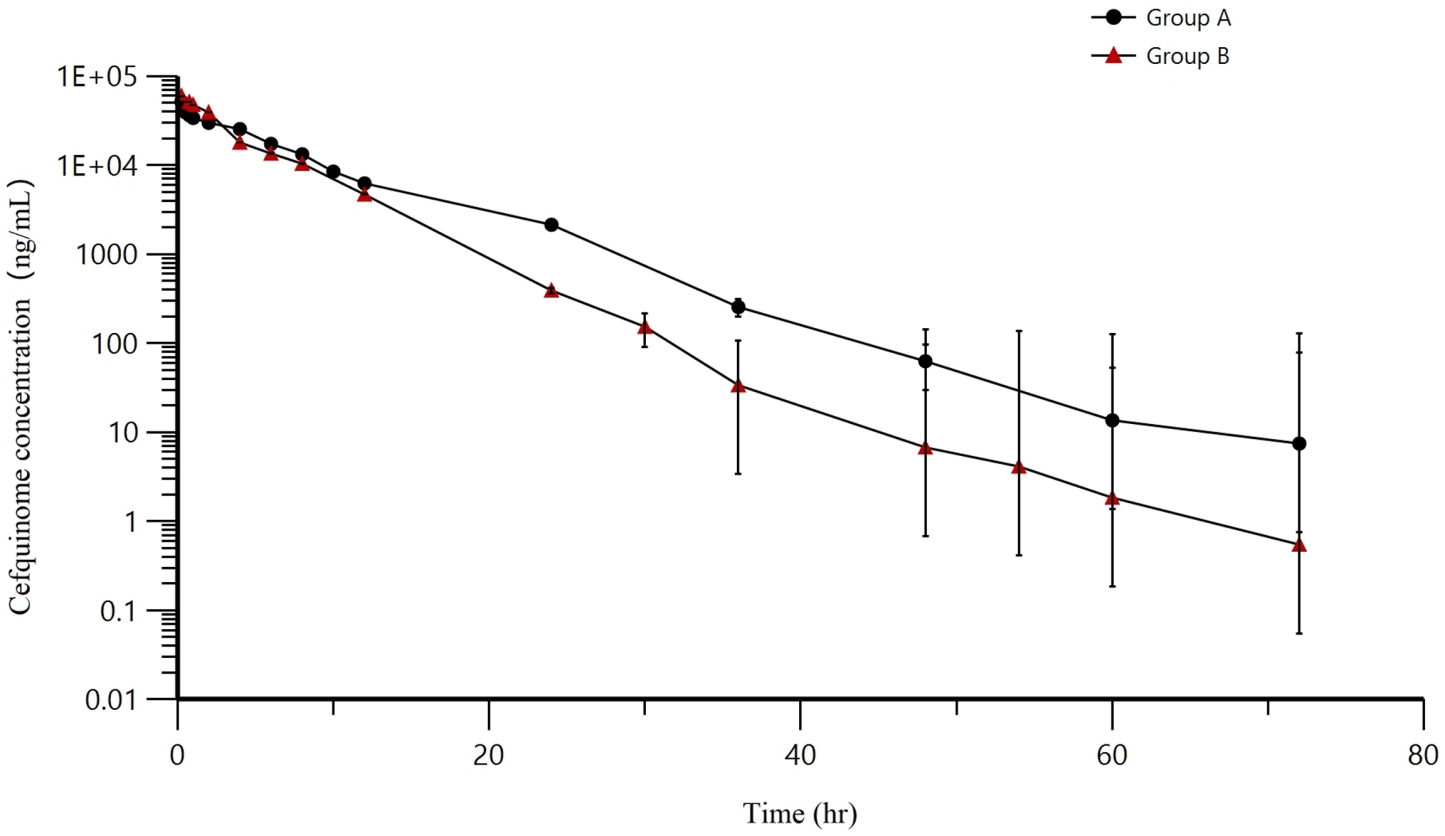
Mean milk concentrations (±SD) in the treated quarters after intramammary administration of cefquinome. Group A = 8 g: 75 mg, Group B = 3 g: 75 mg.

AUCs for two infusions have been illustrated in Figure 5. As time progressed, the concentrations of cefquinome in milk exhibited an exponential decrease following intramammary administration. Throughout the entire research process, no adverse events were found or reported. The AUCs for groups A and B were 300558.57 ± 25052.78 h·ng/mL and 266551.32 ± 50654.84 h·ng/mL, the T_1/2_ for groups A and B were 5.69 ± 0.62 h and 5.25 ± 1.62 h, the MRT for groups A and B were 7.43 ± 0.79 h and 4.81 ± 0.78 h, the Cl/F for groups A and B were 251.14 ± 21.82 mL/h and 290.57 ± 53.98 mL/h, the C_max_ for groups A and B were 51786.35 ± 11948.4 ng/mL and 59763.7 ± 8403.2 ng/mL, and the T_max_ for groups A and B were the same at 0.25 h. Other pharmacokinetic parameters were presented in Table 7.

**Table 7:** Comparison of cefquinome pharmacokinetic parameters and relative bioavailability between Group A (8 g:75 mg) and B (3 g:75 mg).

Parameters	Units	Group A	Group B
AUC_last_	h·ng/mL	300558.57 ± 25052.78	266551.32 ± 50654.84
T_1/2_	h	5.69 ± 0.62	5.25 ± 1.62
λ_z_	1/h	0.12 ± 0.01	0.14 ± 0.05
V_z_/F	mL	2058.95 ± 264.72	2229.57 ± 889.43
Cl/F	mL/h	251.14 ± 21.82	290.57 ± 53.98
MRT	h	7.43 ± 0.79	4.81 ± 0.78
T_max_	h	0.25	0.25
C_max_	ng/mL	51786.35 ± 11948.4	59763.7 ± 8403.2
RBA	%	-	88.69

AUC_last_, the area under the concentration-time curve from zero defining time; T_1/2_, the half-life; λ_z_, the terminal elimination rate constant; V_z_/F, the apparent volume of distribution; Cl/F, the clearance; MRT, mean residence time; T_max_, time to reach C_max_ from time zero; RBA, the relative bioavailability.

## 4 Discussion

In instances of elevated prevalence of *Staphylococcus aureus*-induced mastitis, conventional short-term intramuscular administration of cefquinome may display insufficient pathogen eradication capacities (37). The cure rate of mastitis in cows might possibly get elevated by means of a prolonged regimen of cefquinome administration (38). However, the implementation of such a protocol was constrained by its high cost and impracticality (13). Following the administration of intramammary infusions, the concentration of the drug in the udder will increase rapidly, potentially enhancing the therapeutic efficacy of mastitis treatment (39).

A shortcoming of the multiple dose PK model is that the first sampling time point included in the model may occur after C_max_ and T_max_ (40). It will result in lower reported values for AUC and therefore overestimate reported values for Cl/F and V_z_/F. In addition, repeated administration of cephalosporins may lead to saturation level of the protein binding sites, leading to production of more unbound active metabolites with the passage of time without growing T_1/2_ (41). To avoid this situation, we chose a single dose PK model to study the pharmacokinetics of cefquinome in milk. We obtained the main pharmacokinetic parameters of both formulations using a non-atrial model.

The determination of the half-life holds significant importance within the realms of research and development (42). Following intramammary injection of cefquinome, the half-life (T_1/2_) of cefquinome were 5.69 ± 0.62 h and 5.25 ± 1.62 h in group A (8 g: 75 mg) and group B (3 g:75 mg), respectively. This value differs from single subcutaneous injection of cefquinome into the mouse thigh (T_1/2_ 0.35 h) (43), intravenous injection of cefquinome in horses (T_1/2_ 2.32 h) (44), cow (T_1/2_ 2.12 h) (45) and sheep (T_1/2_ 0.78 h) (46). These differences in data indicate that the variations in half-life of cefquinome are due to different routes of administration and animal species. In clinical practice, the frequency and dosage of administration need to be considered based on the length of the half-life to ensure a consistent and safe concentration of the drug within the body (42). The T_1/2_ of cefquinome in milk obtained from this study can provide a basis for estimating the time required for the drug to reach a steady state in cow milk.

Veterinary Medicines Evaluation Agency of the European Union recommended 75 mg of cefquinome per gland of the under as the cefquinome dosage for treating mastitis (47). Crucially, group B (3 g:75 mg) cefquinome sulfate intramammary infusion significantly decreased the total volume without changing the drug mass. The reduction of infusion volume may protect the mammary gland from mechanical stimulus. After intramammary administration, cefquinome was rapidly absorbed, the peak concentrations (C_max_) observed at 0.25 h were 51786.35 ± 11948.4 ng/mL and 59763.7 ± 8403.2 ng/mL in group A (8 g:75 mg) and group B (3 g:75 mg), respectively. The formula for the infusions, which enhanced the diffusion rate through volume decrease, had been the source of the discrepancy (48). The AUC_last_ were 300558.57 ± 25052.78 h·ng/mL and 266551.32 ± 50654.84 h·ng/mL in group A (8 g:75 mg) and group B (3 g:75 mg), respectively. The AUC depends on the dose of the drug and the rate at which the drug is cleared. In our study, the effective dose of cefquinome was 75 mg/gland in all cases, but the Cl/F in Group A was slower than that in Group B, resulting in a larger AUC.

The FDA established the concept of bioavailability in 1977 (49). Bioavailability is the rate and extent to which the active ingredient is absorbed from a drug product and available at the site of pharmacological action. The AUC method is a faster, cheaper, and more effective way to provide relative values of bioavailability (50). According to the relative bioavailability calculation formula, the RBA is 88.69%, which indicated a high similarity in clinical exposure between two infusions (51). Previous studies have documented that the bioavailability of cefquinome after intramuscular administration was 80.38% (52). The bioavailability of subcutaneous injection of cefquinome >100% (53).Oral bioavailability of cefixime was approximately 80% (54). These cases demonstrates the possibility of bioavailability is closely related to drug dosage forms (55).

Cl/F is a key parameter in pharmacokinetics for assessing the ability of the body to eliminate the drug (56). In the current study, the determined value of Cl/F were 251.14 ± 21.82 mL/h and 290.57 ± 53.98 mL/h for group A (8 g: 75 mg) and group B (3 g: 75 mg), respectively. The results indicate that the cefquinome intramammary infusion of group B (3 g: 75 mg) exhibited a faster drug elimination process compared to group A (8 g: 75 mg). Furthermore, mean residence time(MRT) refers to the duration during which a drug remains in direct interaction with its biological target (57). Previous studies have shown that residence time has been identified as a potential predictor of drug efficacy *in vivo* (58). In the current study, MRT were 7.43 ± 0.79 h and 4.81 ± 0.78 in group A (8 g:75 mg) and group B (3 g:75 mg), respectively. The results suggest that the group B (3 g: 75 mg) exhibited a shorter mean residence time. Thus, the influence of pharmacokinetics should be analyzed based on specific circumstances. The findings of Cl/F suggest the use of Group B infusion has potential for a shorter milk withdrawal period. Milk has important economic benefits for the dairy industry and the general diet, and a shorter withdrawal time is favorable. Shortening the milk withdrawal period reduces milk loss while ensuring food safety of milk to safeguard human health (59).

In this study, extremely minimal cefquinome concentrations (0.43–2.70 ng/mL) were detected in the untreated gland, which is similar to the results of previous research (24). The result indicates that cefquinome exhibit limited penetration across the blood-mammary barrier. This confirms the finding of Rasmussen, who observed little diffusion of cefquinome into the tissue, and indicated that the drug primarily remains within the regions of udder (39). Subsequently, Kietzmann et al. reported that the detected concentrations of cloxacillin within the perfusion samples were below the limit of quantification, which potentially suggests that the absorption of the drug from the regions of udder into systemic circulation was less (60). As is well known, the rumen is an organ containing a large microbial community. Systemic treatment might leads to imbalances in the gut microbiome and destruction of the ecosystem (61). On the contrary, intramammary administration approach prolong duration of drug action in gland and mitigate the common side effects associated with systemic drug infusion (62).

The therapeutic efficacy of β-lactam antimicrobials was decided by the duration of exposure of infectious agents to concentrations above the minimum inhibitory concentration (63). Cefquinome exhibited a time-dependent killing that exhibit bactericidal effects when the concentration in the target-controlled organ exceeded the pathogen’s minimum inhibitory concentration (MIC) (64, 65). Studies have shown that MIC of *Staphylococcus aureus*, *Streptococcus species*, and *Escherichia coli* isolated from cows were 0.5–1 μg/mL, 0.25–0.5 μg/mL, and 0.06–0.13 μg/mL, respectively (46). In the current study, cefquinome showed sustained concentrations in gland above 1 μg/mL for 12 h. Therefore, dosage regimen of 75 mg/gland with 12 h intervals should be appropriate when cefquinome sulfate intramammary Infusion used in cows for the treatment of mastitis.

In conclusion, our study established an UPLC-MS/MS method to study the pharmacokinetics of two cefquinome sulfate intramammary infusions. Two infusions exhibit similar pharmacokinetic behavior and the RBA is 88.69% and share identical dosage regimen. This study provides valuable information for veterinary clinical medication and decision making.

## Data availability statement

The original contributions presented in the study are included in the article/Supplementary material, further inquiries can be directed to the corresponding authors.

## Ethics statement

The animal studies were approved by the Animal Use and Care Committee of the Feed Research Institute, Chinese Academy of Agricultural Sciences (IFR-CAAS20230309). The studies were conducted in accordance with the local legislation and institutional requirements. Written informed consent was obtained from the owners for the participation of their animals in this study.

## Author contributions

SL: Writing – original draft, Writing – review & editing, Methodology, Software, Validation. NY: Methodology, Software, Validation, Writing – original draft, Writing – review & editing. YT: Data curation, Formal analysis, Methodology, Software, Writing – review & editing. CL: Conceptualization, Writing – original draft. YZ: Conceptualization, Writing – original draft. XC: Formal analysis, Investigation, Writing – original draft. HW: Data curation, Resources, Visualization, Writing – original draft. XL: Funding acquisition, Project administration, Writing – original draft, Writing – review & editing. YL: Funding acquisition, Project administration, Supervision, Writing – review & editing.
