# Supplementary material for: Pharmacokinetics and relative bioavailability study of two cefquinome sulfate intramammary infusions in cow milk
Source: Front Vet Sci. 2024 Mar 11;11:1384076. doi: 10.3389/fvets.2024.1384076 (PMC10962211; doi:10.3389/fvets.2024.1384076)
Supplement: Supplementary file 1 [file Data_Sheet_1.docx]

Supplementary Material

# Supplementary Figures

**
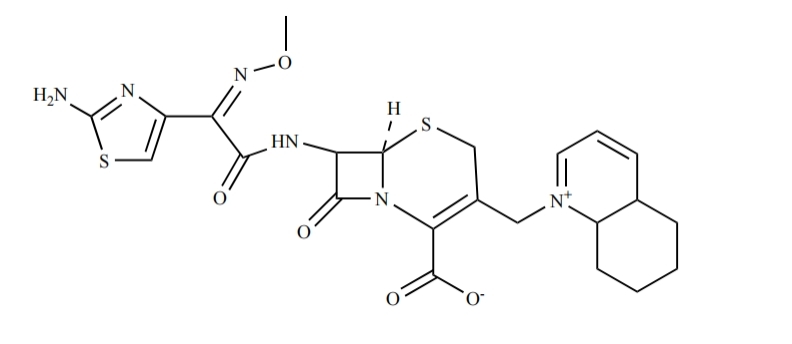
**

**Supplementary Figure 1.** The chemical structural formula of cefquinome.


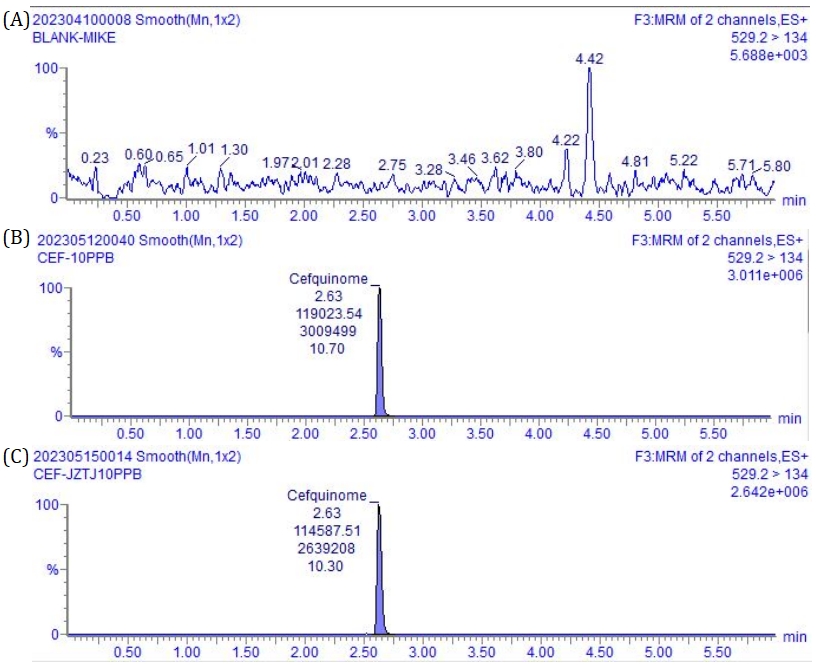


**Supplementary Figure 2.** UPLC-MS/MS chromatograms. **(A)** The chromatogram of blank milk sample **(B)** The chromatogram of blank milk sample added cefquinome (10 ng/g) **(C)** The chromatogram of blank mobile phase added cefquinome (10 ng/mL)


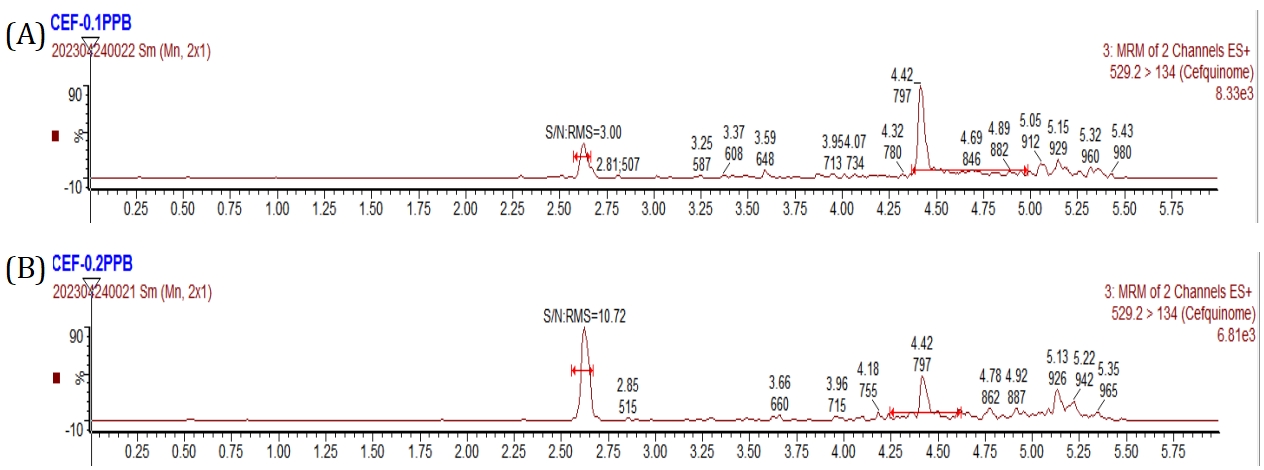


**Supplementary Figure 3.** UPLC-MS/MS chromatograms. **(A)** The chromatogram of LOD for cefquinome **(B)** The chromatogram of LOQ for cefquinome


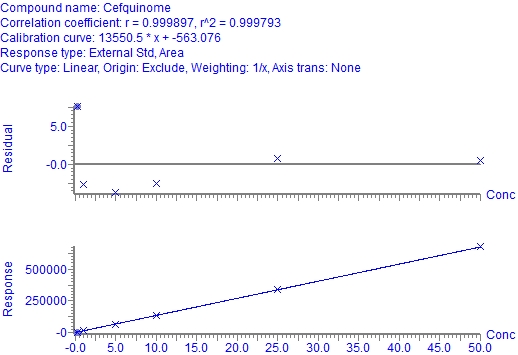


**Supplementary Figure 4.** Calibration curve for cefquinome in cow milk

.


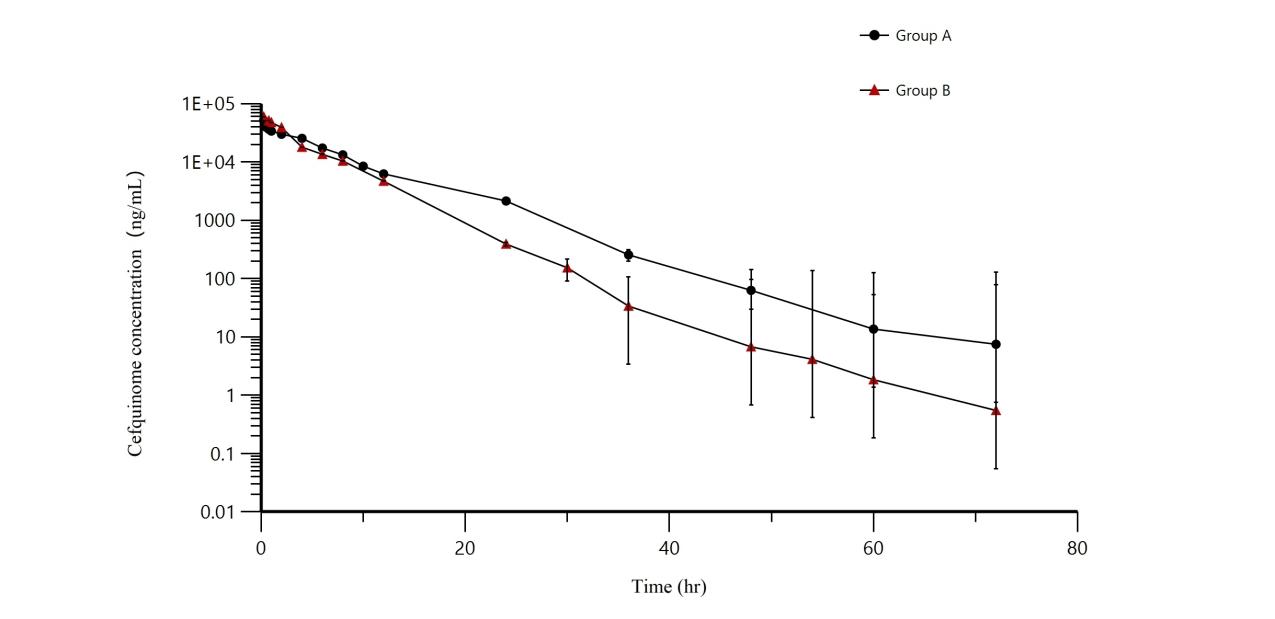


**Supplementary Figure 5.** Mean milk concentrations (± SD) in the treated quarters after intramammary administration of cefquinome. Group A=8 g: 75mg, Group B=3 g: 75mg.

**Table 6.** Stability of cefquinome under various storage conditions.

| **Spiked concentration（ng/g）** | **reserve solution**  **(30 days at -40°C)** | **freeze-thaw** | **sample tray**  **(8h at 4°C)** | **Long-term stability (90 days at -40°C)** |
| --- | --- | --- | --- | --- |
|  | **Mean ± SD** | **Mean ± SD** | **Mean ± SD** | **Mean ± SD** |
| 0.2 |  | 0.21±0.02 | 0.21±0.01 | 0.20±0.02 |
| 10 | 10.32±6.67 | 8.35±0.25 | 10.32±0.52 | 8.5±0.16 |
| 20 |  | 19.71±1.04 | 19.85±1.06 | 20.56±0.88 |
| 40 |  | 38.82±1.94 | 40.19±1.64 | 39.92±2.18 |
